# The transcriptional regulation of the human angiotensinogen gene after high-fat diet is haplotype-dependent: Novel insights into the gene-regulatory networks and implications for human hypertension

**DOI:** 10.1371/journal.pone.0176373

**Published:** 2017-05-03

**Authors:** Anita Rana, Sudhir Jain, Nitin Puri, Meenakshi Kaw, Natalie Sirianni, Deniz Eren, Brahma Raju Mopidevi, Ashok Kumar

**Affiliations:** 1 Department of Physiology and Pharmacology, University Of Toledo Medical Centre, Toledo, Ohio, United States of America; 2 Department of Pathology, New York Medical College, Valhalla, New York, United States of America; Wayne State University, UNITED STATES

## Abstract

Single nucleotide polymorphisms (SNPs) in the human angiotensinogen (hAGT) gene may modulate its transcription and affect the regulation of blood pressure via activation of the renin-angiotensin aldosterone system (RAAS). In this regard, we have identified polymorphisms in the 2.5 Kb promoter of the hAGT gene that form two haplotype (Hap) blocks: -6A/G (-1670A/G, -1562C/T, -1561T/C) and -217A/G (-532T/C, -793A/G, -1074T/C & -1178G/A). hAGT gene with Hap -6A/-217A (Hap I) is associated with increased blood pressure whereas, Hap -6G/-217G (Hap II) is associated with normal blood pressure in human subjects. Since RAAS over activity contributes to hypertension in obesity, we have made transgenic mice (TG) containing either Hap I or Hap II of the hAGT gene to understand the role of obesity on its transcriptional regulation. Although, a high-fat diet (60% Kcal from fat, 12 weeks) elevates hAGT and mAGT regardless of haplotype, this effect is significantly (p<0.05) accentuated in Hap I mice, in both adipose and liver tissues. Chromatin Immuno- precipitation (ChIP) assay shows an increased binding of transcription factors including, GR, CEBPβ and STAT3 to the chromatin of the Hap I TG mice after high-fat diet as compared to Hap II TG mice (p<0.05). Differential plasma levels of hAGT in Hap II and I mice, after high-fat diet, further corroborate the variable transcriptional regulation of the hAGT, governed by gene-haplotypes. Taken together, our results show that SNPs in the Hap-I of the hAGT gene promote high-fat diet-induced binding of transcription factors GR, CEBP-β and STAT3, which lead to elevated expression of the hAGT gene in hepatic and adipose tissues.

## Introduction

Hypertension is a complex disorder arising from intricate crosstalk between environmental factors and genetic predispositions [1, 2]. Importantly, the genetic makeup of a subject can greatly influence the impact of a particular environmental stimulus such as high-fat diet or high-salt diet. For example, although obesity and hypertension frequently co-existent, every obese individual is not hypertensive [3, 4]. In this regard, we have focused our work on groups of single nucleotide polymorphism (SNPs) in genes relevant to the regulation of mammalian blood pressure. Although human angiotensinogen (hAGT) gene is associated with hypertension, its transcriptional regulation in pathological scenarios like obesity is poorly understood.

AGT is the sole substrate of the renin-angiotensin system (RAS), which is central to mammalian blood pressure regulation [5–8]. RAS over-activity is one of the causes of human hypertension that leads to increased risk of stroke and myocardial infarction [6, 9]. Many reports have established a positive correlation between plasma AGT levels and blood pressure in humans and experimental animal models [10, 11]. The role of AGT gene in hypertension is also suggested by studies that showed elevated plasma AGT level by increasing AGT gene-copy number and an increase in blood pressure in TG mice [12, 13]. Therefore, we have used TG-mice containing different haplotypes of the hAGT gene to understand the effect of different SNPs on transcriptional regulation and blood pressure regulation in an *in vivo* setting.

The human AGT gene contains multiple SNPs in its 2.5 Kb promoter [14–16]. Many of these SNPs are in linkage disequilibrium (LD) and always occur together [17–19]. We have shown that SNPs in the -6A/-217A sub-block (Hap I) confer increased risk of hypertension whereas, the -6G/-217G sub-block (Hap II) is protective [19, 20]. However, role of these haplotype, if any, in the AGT gene-regulation during environmental stress is unknown. Diet induced obesity is one of such stress. How specific genetic components contribute to the obesity-related hypertension is still unclear. Thus, we have hypothesized that the chronic oxidative/ inflammatory milieu of obesity will alter cellular transcriptional machinery that, in turn, will have haplotype-dependent effect on the AGT expression. Using single transgenic mice of either haplotype [20], we show here that high-fat diet (HFD) up-regulates hAGT gene expression significantly more in Hap I TG mice. This effect is due to higher affinity of transcription factors to the chromatin of Hap I hAGT TG mice as compared to the chromatin of Hap II TG mice.

## Results

### High-fat diet (HFD) increases hAGT gene expression in adipose and liver of Hap I TG mice

**Fig 1:**
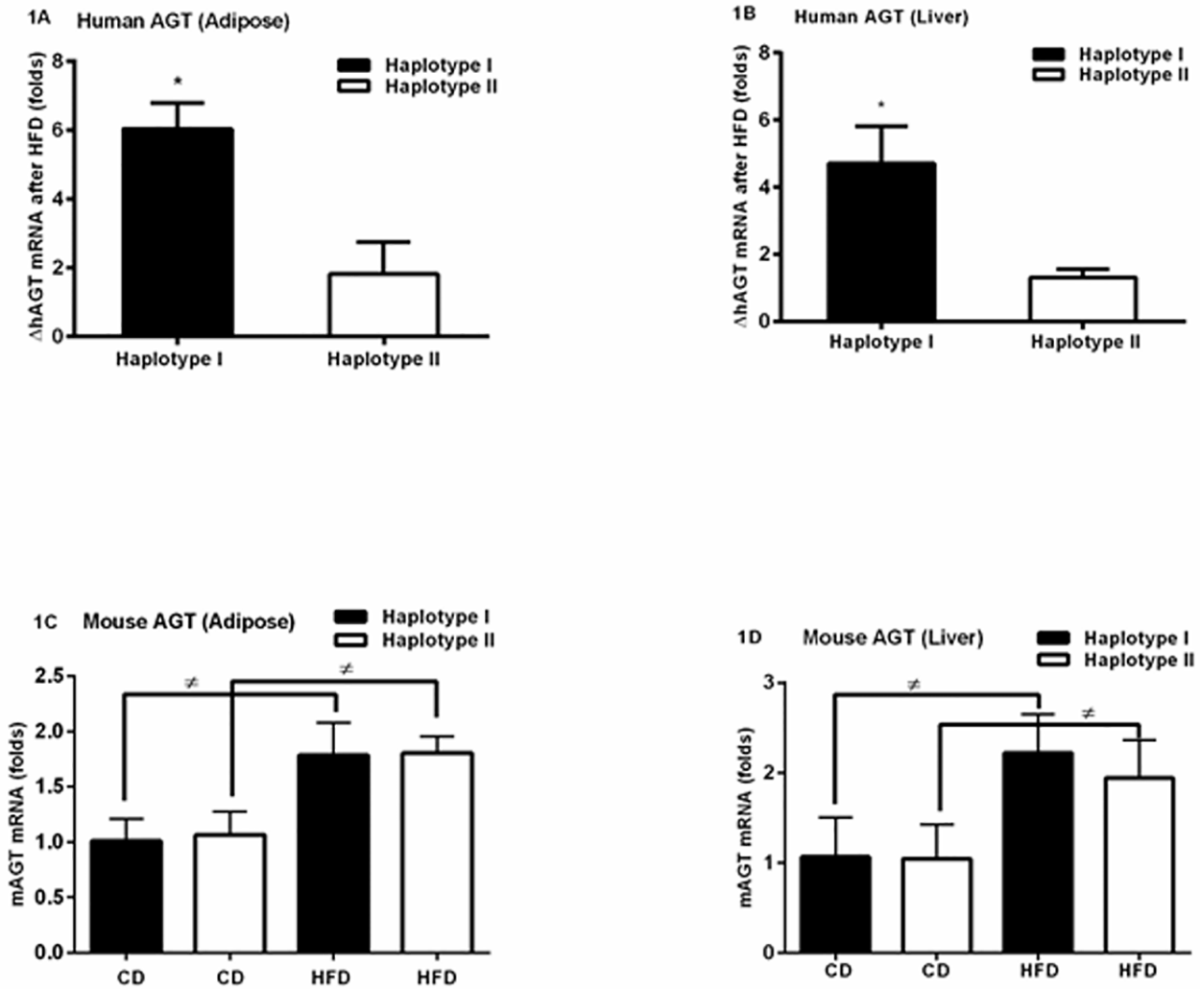
TG-mice with Hap I have increased expression of the hAGT gene after HFD, as compared with Hap II. Human AGT expression is significantly elevated after HFD in TG mice with Hap I than Hap II, in adipose and liver tissues. Change in mRNA expression of the hAGT gene after 12 weeks of HFD, as compared to baseline CD, in adipose (A) and liver tissue (B). mAGT expression in adipose (C) and liver tissue (D) of TG mice fed with control diet (CD) or HFD in both haplotypes. mRNA was determined by quantitative RT-PCR analysis. Results are shown as mean ±SEM (error bars) from n = 4 per group. *p ≤ 0.05 *versus* Hap II with HFD; ^#^ p ≤0.05 *versus* CD in both Hap I & Hap II.

We tested the effects of the HFD on the transcriptional regulation of different haplotypes of the hAGT gene. High-fat diet up regulated the hAGT expression in both groups; however, change in the hAGT expression is significantly (p<0.05) greater in adipose (Fig 1A) and liver (Fig 1B) (p ≤ 0.05) tissues in Hap I as compared to Hap II TG mice (Data points shown in S1 Fig). Although, HFD also increases the endogenous mAGT gene expression at the mRNA level, this effect was independent of haplotype (Fig 1C and 1D) (Data points shown in S1 Fig and S1 File).

### High-fat diet increases the expression of transcription factors involved in expression of the hAGT gene

**Fig 2:**
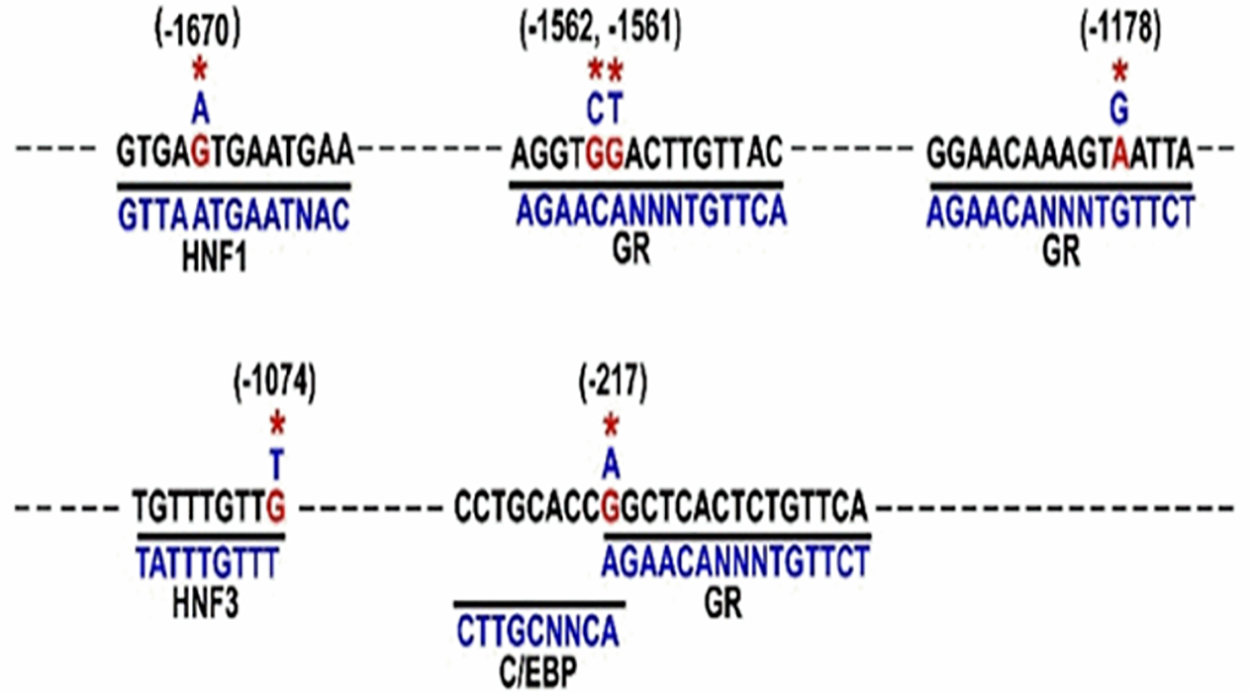
Transcription factor binding sites in the hAGT gene promoter. Nucleotide sequence of different regions of the promoter of the hAGT gene along with position of SNPs (marked by asterisks). Variants in Hap II are shown in red, and in Hap I are shown in black. Consensus binding sites of different transcription factors are shown below the nucleotide sequence of the promoter.

**Fig 3:**
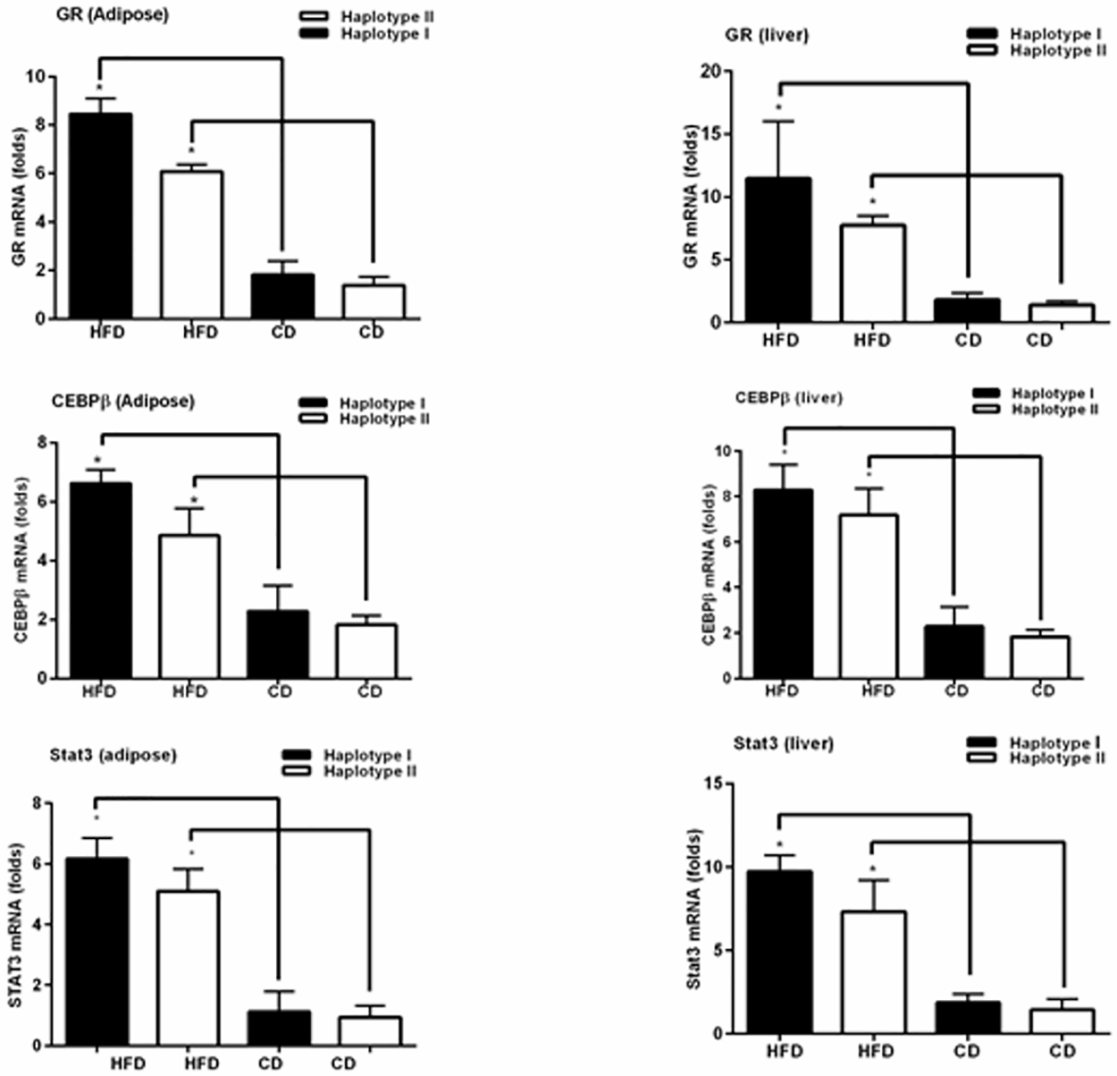
Expression of the transcription factors associated with the regulation of the hAGT, with or without HFD. Expression of transcription factors, GR, CEBPβ, and STAT3 with or without HFD in adipose (A) and liver (B) tissue. Expression of mRNA was calculated for the HFD and CD group and normalized by the respective GAPDH values. Results are shown as mean ± S.E. (error bars) (n = 4). *p ≤0.05 versus Hap I & Hap II with CD.

*In silico* Transfac analysis shows that nucleotide sequence present in the promoter of Hap I of the hAGT gene has stronger homology with consensus binding sites of transcription factors GR, CEBPβ, HNF3 and STAT3 as compared to Hap II of the hAGT gene (Fig 2). Therefore, we measured the expression of CEBPβ, STAT3 and GR in adipose and liver of adult male Hap I and Hap II TG mice after high fat diet by Q-RT-PCR. Results of these experiments show that mRNA levels of CEBPβ, STAT3 and GR were significantly (p ≤0.05) increased in adipose (Fig 3A) and liver (Fig 3B) after HFD, regardless of the haplotype (Data points shown in S2 Fig and S1 File).

### High-fat diet increases binding of transcription factors to the promoter and enhancer of the hAGT gene in Hap I TG Mice as compared to Hap II

**Fig 4:**
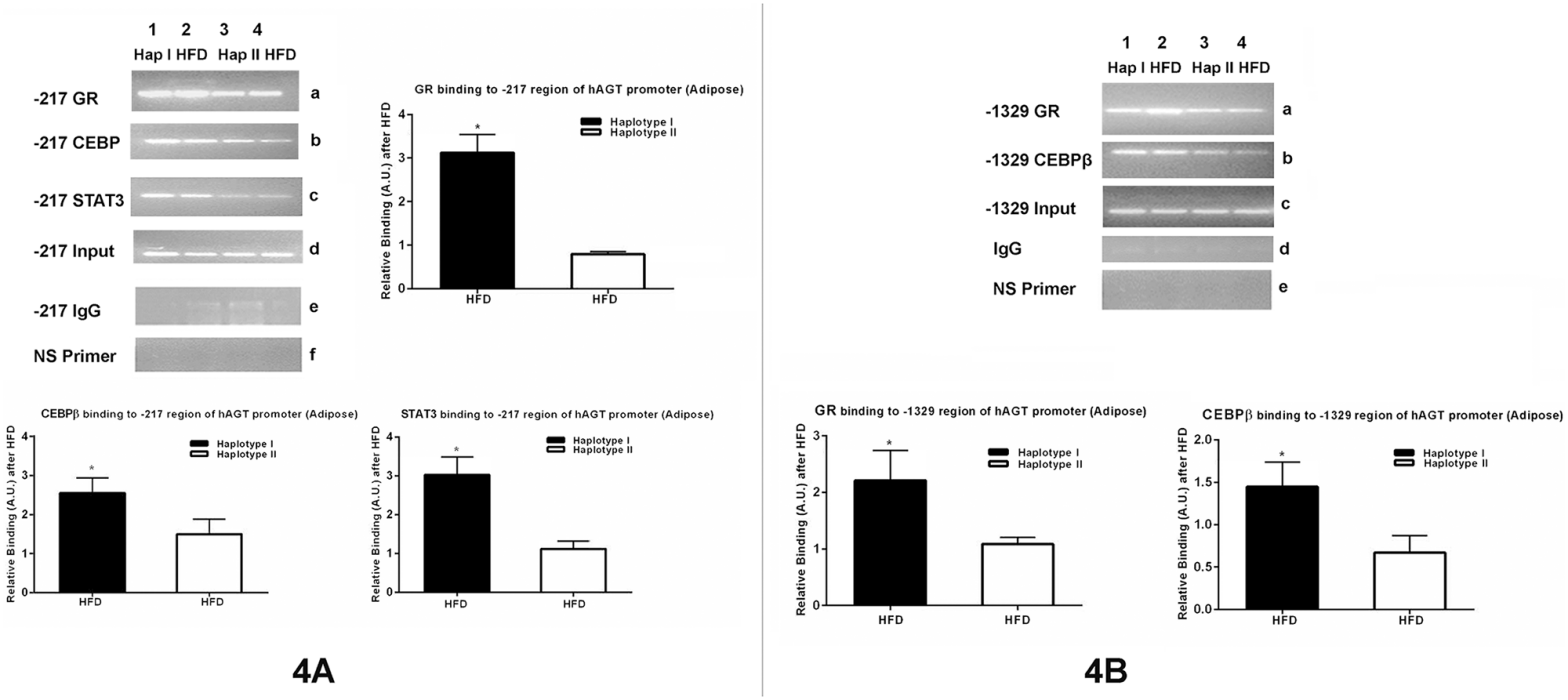
ChIP assay on the -217 and -1329 regions of the hAGT gene from the chromatin obtained from adipose tissue of TG mice after HFD. ChIP assay was performed by PCR amplification of the immunoprecipitated DNA in the presence of antibodies against GR (a), CEBPβ (b) and STAT3 (c), input DNA (d), IgG (e), and nonspecific primers (NS) (f). Immunoprecipitated DNA was used to amplify nucleotide sequence encompassing either -217 region (Fig 4A) or -1329 region (Fig 4B) of the hAGT gene as described in “Materials and Methods.” Quantitation of GR, CEBPβ and STAT3 -enriched DNA, relative to input, at the -217 region or -1329 region of the hAGT gene was performed by Q-PCR. Result shows a significant increase in the HFD-induced GR, CEBPβ and STAT3 binding in TG mice with Hap I as compared to Hap II. *, p≤0.05 *versus* Hap II with HFD. Results are shown as mean ± S.E. (n = 4). A.U., arbitrary units.

Since SNPs at the nucleotide position, -217 and -1561/-1562 in the Hap I TG mice have stronger homology with the consensus binding sites of GR, STAT3 and C/EBPβ; we examined the effect of the HFD on the binding of these transcription factors to the -217 and -1561/-1562 regions of the hAGT gene promoter. A ChIP assay was performed using GR, CEBPβ and STAT3 specific antibodies in the presence of chromatin from adipose tissue of the TG animals. As shown in Fig 4A, GR, C/EBPβ, and STAT3 bind more strongly to the chromatin obtained from the adipose tissues of the HFD-treated Hap I TG mice, as compared with HFD-treated Hap II. Similarly, GR and C/EBPβ binding to the upstream promoter of the hAGT was also tested (Fig 4B). As with the -217 region, GR and C/EBPβ bind much more strongly (p<0.05) to the upstream region of the promoter obtained from HFD-treated Hap I mice as compared to similarly treated Hap II mice (Data points shown in S3 and S4 Figs respectively and in S1 File).

### HFD increases plasma hAGT in Hap I TG mice

**Fig 5:**
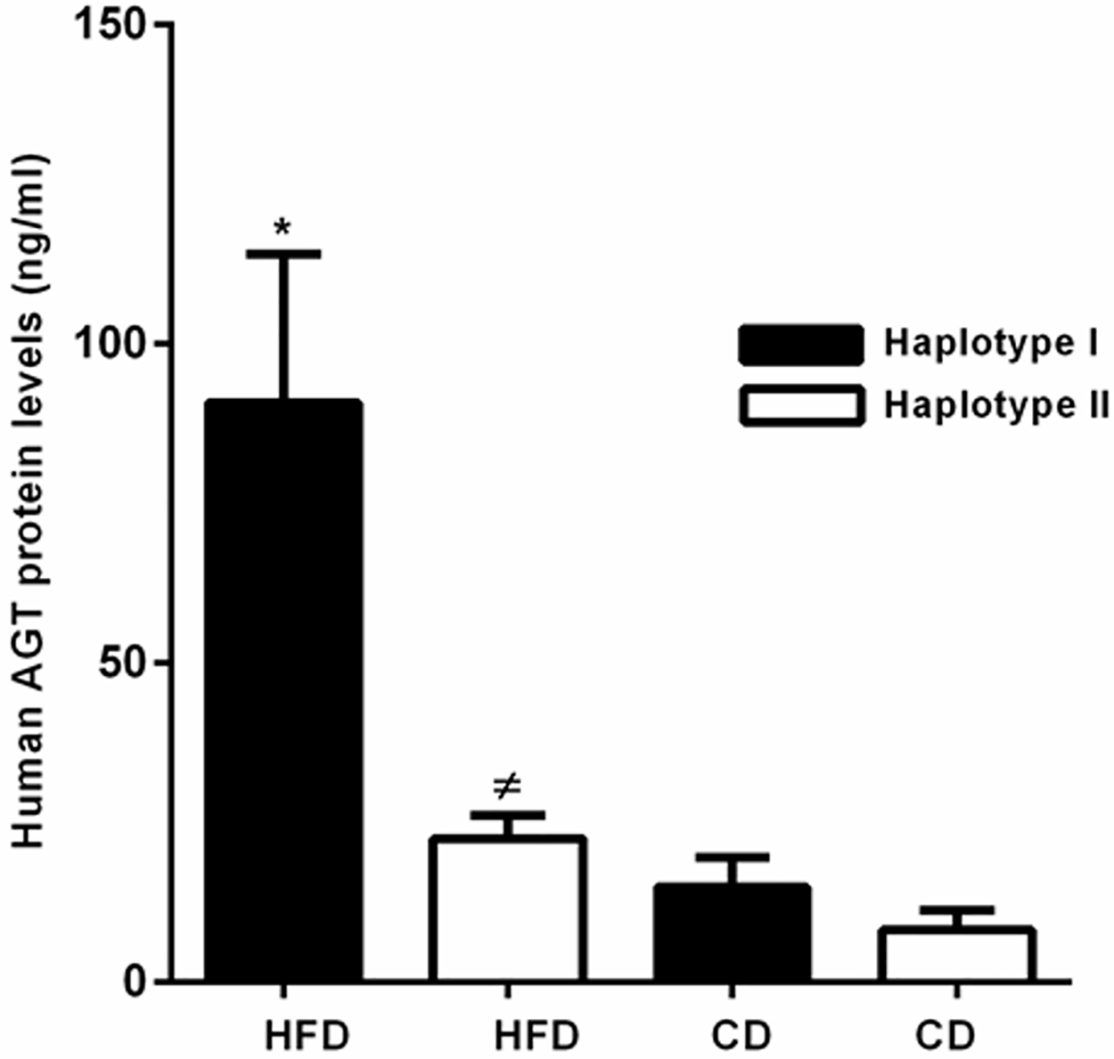
Effect of HFD on plasma level of hAGT in Hap I and Hap II TG mice. Plasma levels of hAGT in TG mice containing either Hap I or Hap II of the hAGT gene in CD or HFD fed TG mice. Results are shown as mean ± SEM from n = 4. *p≤0.05 versus Hap II with HFD; # p≤0.05 versus Hap II with CD.

In order to determine the systemic implications of increased tissue AGT, caused by HFD, we examined hAGT in plasma of TG mice by ELISA. Baseline plasma hAGT is significantly (p ≤0.05) increased in Hap I TG mice as compared to Hap II (Fig 5). Moreover, HFD significantly (p≤0.05) increases the plasma hAGT level only in Hap I TG mice and not in Hap II TG mice (Data points shown in S5 Fig and S1 File).

## Discussion

In the present study, we have examined the HFD-mediated, allele-specific, transcriptional regulation of the hAGT gene in adipose and liver tissues. Previous studies have suggested that the hAGT gene contains 4 major haplotypes. Hap I (containing variants -1670A, -1562C, -1561T, -6A, -217A, -532T, -793A, -1074T, and -1178G) has the highest risk for increased blood pressure whereas, the Hap II (containing variants -1670G, -1562G, -1561G, -6G, -217G, -532C, -793G, -1074C, and -1178A) confers the lowest risk for increased blood pressure [14]. To understand the role of these haplotypes of the hAGT gene in hypertension, and other cardiovascular complications, we have made TG mice containing either Hap I or Hap II of the hAGT gene. We have previously shown that TG mice with Hap I have increased blood pressure as compared to Hap II [20]. Using these novel TG mice, we show here, for the first time, that HFD alters the transcriptional milieu of cells with up-regulation of the hAGT gene. Importantly, this effect is AGT-haplotype-dependent and is more pronounced in Hap I TG mice.

RAAS over-activity and hypertension are frequent accompaniments of diet-related pathologies like, the metabolic syndrome. It is well accepted that the liver and the adipose tissues are the primary source of systemic AGT in rodents and humans. We have previously shown that the hAGT gene expression is higher in liver and fat of the Hap I TG mice as compared to the Hap II [20].**The first key finding of this study** is that HFD positively regulates the hAGT expression, which is greater in mice with HAP I. Higher hAGT expression in adipose and liver tissue is observed after HFD in both Hap I and Hap II TG mice. However, difference in hAGT expression was much higher in the Hap I as compared to the Hap II TG mice. These results are exciting but not unexpected since other investigators have previously reported diet-induced induction of the AGT in liver and adipose tissues [21–24]. This is the first report, however, showing that SNP differences in the haplotypes govern the response of the gene to a physiological scenario like, diet. Haplotype-independent response of the endogenous mAGT to diet further supports this notion that promoter SNPs can have significant impact on gene transcription. These findings are highly relevant as over—expression of the AGT gene in adipose tissue has been shown to increase adipose mass and elevate systemic AGT concentration [25].

**The second key finding of this study**, in agreement with published reports, is the diet-induced increase in levels of transcription factors in the liver and the adipose tissues. Expression of glucocorticoid receptors, STAT3 and CEBPβ is significantly increased by HFD. It is noteworthy that this increase in transcription factors is haplotype-independent. Transcription of a gene is regulated by a plethora of transcription factors, binding to their cis-acting DNA elements in the promoter and enhancer regions of a gene. SNPs in the promoter and/or enhancer regions of the gene alter this binding and thus, have the potential to modulate gene transcription. Nucleotide sequence of the hAGT gene promoter containing -217A (Hap I) has stronger homology with the consensus C/EBPβ binding site as compared to -217G. C/EBP plays an important role in the expression of a gene in the hepatocytes [26–28] and the adipocytes [29]; two cell types in which the AGT gene is primarily expressed. In addition, C/EBP-β and -δ are involved in IL-6 and TNFα-induced expression of various genes [30]. Surprisingly, a glucocorticoid responsive element is also located at this site and our previous ChIP assay has shown that GR binds more strongly to the hAGT promoter sequence containing -217A [16]. Previous work has also shown that binding of C/EBPβ acts like a “pioneer” transcription factor and opens up the chromatin to allow the binding of the GR to the opened chromatin [31]. In addition, nucleotide sequence at the -217-region of the promoter in the Hap II TG mice is CCGC (Fig 2) and contains a dinucleotide CG, which may be methylated. As a result, methylated C^Me^G will inhibit the binding of the transcription factors to this site in the Hap II TG mice. On the other hand, nucleotide sequence of the promoter region of the hAGT in the Hap I TG mice contains CCAG (Fig 2), which cannot be methylated and therefore transcription factors can bind to this sequence. Since GR and C/EBP bind more strongly to the nucleotide sequence in the promoter in the Hap I TG mice, it will result in increased transcription of the hAGT gene in the Hap I TG mice. Previous genome-wide ChIP analysis by Choy *et al*. has shown that conserved consensus transcription factor binding sites are hyper-methylated in the genome and this methylation inhibits the binding of the transcription factors [32]. This genome wide analysis supports our results that the transcription of the hAGT gene will be increased in Hap I TG mice containing nucleotide sequence CCAG as compared to Hap II, as that contains nucleotide sequence CCGC.

Nucleotide sequence of the hAGT gene around -1074 has a HNF3 binding site (TA/GTTTA/GTTT). Interestingly, variant -1074T (present in the Hap I) makes stronger HNF3 binding site as compared to variant -1074G (present in the Hap II). Transcription factor HNF3 plays an important role in the liver-specific expression of a gene. In addition, HNF3 has been termed as the “pioneer” transcription factor and opens up the chromatin for binding with the estrogen, androgen and mineralocorticoid receptors [33, 34]. Additionally, the nucleotide sequence of the hAGT gene containing the variant -1178G has stronger homology with GRE as compared to the promoter sequence containing -1178A. In addition, nucleotide sequence of the hAGT gene containing variant -1561T and -1562C has stronger homology with the GRE. It is worth mentioning that the expression of GR, CEBPβ, and STAT-3 is significantly increased by HFD.

This leads to the **third important finding of this study**; HFD-altered transcriptional milieu modulates the human AGT expression in a haplotype-dependent manner via enhanced transcription factor binding to the chromatin of the hAGT in the HAP I mice. Expression of these transcription factors is independent of haplotype but, we have found an increased binding of GR and CEBPβ to the chromatin obtained from the adipose tissue of the Hap I TG mice as compared to Hap II. As discussed earlier, SNPs in the promoter of Hap I of the hAGT gene have stronger binding with HNF3 and C/EBP-β [20]. Importantly, it is known that GR interacts with C/EBP-β and HNF3 in regulating the expression of multiple genes in the liver cells [28, 35–37]. Thus, our data shows that the HFD increases the expression of multiple transcription factors that ultimately result in increased expression of the hAGT gene containing Hap I via variable, haplotype-dependent binding of transcription factors to the hAGT gene in Hap I TG mice.

Diet-induced obesity is a chronic inflammatory state [24, 25]. This sub-clinical inflammation has been shown to modify the cellular transcriptional milieu including, tissue levels of glucocorticoids and STAT3 [38]. The human AGT is an acute phase protein and its expression is increased in inflammation mainly through IL-6 pathway. Sherman et al. have shown that AGT expression is increased in HepG2 cells by IL-6 treatment and have identified three signal transducers and activators of transcription (STAT-3) binding sites (APRE1 located between -269 and -278; APRE2 located between -237 and -246; and APRE3 located between -162 and -171) [38]. We have shown previously that transient transfection of a reporter construct containing only 223 bp of the 5-flanking region of the hAGT gene (which contains the APRE3 site) is trans-activated by IL-6 treatment and showed that STAT-3 interacts with C/EBP- β, HNF-1 and GR to regulate transcription of the hAGT gene. Since STAT-3 also physically interacts with CEBPβ [38, 39], interaction of STAT-3, HNF-1α, GR, CEBPβ and CBP may be responsible for IL-6 induced expression of the hAGT gene [40].

We observe an increased binding of CEBP-β and STAT3 after HFD diet in chromatin obtained from the adipose tissue of Hap I TG mice. Since endogenous mAGT gene does not contain SNPs to modulate GR, or C/EBP- β binding, we did not observe differential regulation of the mAGT gene by HFD, which lends credibility to the hypothesis that HFD promotes haplotype-mediated transcription of the hAGT gene. Thus, these results strongly suggest that the transcriptional milieu of liver and adipose tissues is altered after HFD, which then affect the hAGT expression in a haplotype-dependent manner due to, increased affinity of the chromatin of the Hap I TG mice to transcription factors such as CEBP-β, GR, HNF-1 and STAT3. It is important to mention in this context that Friese *et al*. have measured the expression of different genes in the adrenal gland of hypertensive rats and mice (SHR, WKY, blood pressure high mouse BPH, and blood pressure low mice BPL) by microarray and found that CEBPβ expression is increased in hypertensive animals [41]. Earlier studies have also suggested that transcription factors STAT3 and CEBPβ can undergo post-translational phosphorylation by multiple protein kinases [42, 43]. This phosphorylation increases the DNA binding activity of STAT3 and CEBPβ that ultimately results in increased transcription of a gene.

Systemic implications of increased transcription of the hAGT gene by HFD (HFD) in Hap I TG mice were confirmed by the assessment of plasma AGT levels. These observations are supported by previous studies that demonstrated the activation of the systemic RAAS in experimental models of diet-induced obesity [44–46]. It was also noted that rats fed with moderately high-fat (HF) diet exhibited increased plasma angiotensin II (Ang II) concentrations and elevated systolic blood pressures (SBPs) [47].

## Conclusions and perspectives

To summarize, pathophysiological variables like the HFD alter the cellular transcriptional milieu that ultimately results in the modulation of the hAGT gene expression. HFD increases the expression of transcription factors such as GR, HNF-1, STAT-3 and C/EBPβ in the liver and adipose tissues, which results in increased binding of these transcription factors to the chromatin and increased expression of the hAGT gene in animals containing Hap I of this gene. This would potentially make subjects with Hap I more susceptible to HFD-induced, AGT-mediated complications such as hypertension. Clinical significance of this study is that it will help identify individuals with risk haplotype undergoing diet induced metabolic syndrome. In this setting, identification of “at-risk” haplotypes for the hAGT will benefit patients by providing timely and targeted therapy so as to prevent long-term complications. It is worth noting that the sample size in this study is small due to low-breeding of the TG lines but, the data remains significant nonetheless.

## Material and methods

### Transgenic (TG) mice and diets

All animal experiments were performed according to the National Institutes of Health Guide for the Care and Use of Laboratory Animals and approved by the institutional ethical animal care and use committee at the University of Toledo College of Medicine (UTMC). The human AGT (hAGT) TG mice utilized in this study were generated in our laboratory as described previously [20]. Genotyping analysis of the tail snips, followed by sequencing, was performed to confirm the genetic lineage of these TG mice. As described previously, the TG mice with Hap I have variants -6A, -20A, -217A, -532T, -793A, -1074T, -1178G, -1561T, -1562C, and -1670A; whereas, TG mice with Hap II have variants -6G, -20A, -217G, -532C, -793G, -1074G, -1178A, -1561G, -1562G, and -1670G. As described previously, we have performed copy number analysis on all our transgenic lines and, although the gene-insertion point is unknown, all mice used in the described experiments have a single copy of the hAGT transgene [20]. For experimental purposes, these TG mice were housed individually. Twelve-week-old TG mice containing Hap I or Hap II were divided into four groups (n = 4). At study end point, mice were anesthetized with ketamine and xylazine (100/10 mg/kg, IP) for exsanguination and tissue harvesting. Male mice of each haplotype were fed either a control diet (CD) (10% kcal as fat; D12450B; Research Diets Inc, New Brunswick, NJ) or a HFD (60% kcal as fat; D12492, Research Diets Inc, New Brunswick, NJ) for 12 weeks. Diets were matched in protein content (20% kcal) and provided energy at 3.85 or 5.25 kcal/gm (control diet and HF diet, respectively). Diets were fed to mice *ad libitum*. Animals were maintained in a 22°C room with a 12-h light/dark cycles and received drinking water *ad libitum*.

### Quantification of plasma human AGT

Plasma hAGT levels were determined by an ELISA kit purchased from Ray Biotech, Inc in male TG-mice. The hAGT concentration in the samples was determined directly from the standard curve according to the manufacturer’s protocol [20].

### Tissue RNA extraction and quantitative RT-PCR

Adipose and liver tissue were harvested at the end of the experiments and snap-frozen in liquid nitrogen. The extracted tissues were stored at -80°C until utilized for further experiments. RNA was isolated using RNeasy Plus mini kit (Qiagen). RNA (1ug) was reverse-transcribed into cDNA using the Revert Aid first strand cDNA synthesis kit (Fermentas), as described in the protocol. Following a 95°C incubation for 10 min, 40 cycles of PCR (95°C for 30s, 60°C for 30s), were then performed using 1μl of cDNA, 50 nM PCR primers, and 12.5 μl of SYBR Green PCR Master Mix in 25μl reactions. Threshold cycles for three replicate reactions were determined using MxPro-Mx3005P software (version 4.10), and relative transcript abundance was calculated following normalization with mouse GAPDH. Primers for human and mouse AGT and mouse GAPDH were obtained from Super Array Bioscience Corp. (Frederick, MD) or from Integrated DNA Technologies (Coralville, IA). In general, relative quantification relates the PCR signal of the target transcript in a treatment group to that of another sample, such as untreated control. This is a widely used method to present relative gene expression by the comparative CT method, also referred to as the 2^-ΔΔCT^ method [47–49].

### In vivo chromatin immunoprecipitation (ChIP) assays

The ChIP assay was performed using the EZ-ChIP assay kit from EMD Millipore as described previously [16]. Mice were perfused with normal saline, and the adipose tissues were removed, washed, and fixed with 1% formaldehyde for 20 min at room temperature. The DNA was fragmented by sonication, and 10μl of the chromatin solution was saved as input. 5 μg of anti-glucocorticoid receptor (anti-GR) and anti CEBPβ (anti-CEBPβ) or rabbit immunoglobulin G were added to the tubes containing 900 μl of sonicated chromatin solution; the mixture was incubated overnight at 4°C. The antibody complexes were captured with the protein A-agarose beads and subjected to serial washes (as described in the manufacturer’s protocol). The chromatin fraction was extracted with SDS buffer and reverse cross-linked at 65°C for 4–6 h. The DNA was then purified as described in the manufacturer’s protocol. The immuno-precipitated DNA (1 μl) and the input DNA (1μl) were subjected to 35 cycles (of PCR amplification (denaturation at 95°C for 30 s, annealing at 58°C for 30s, extension at 72°C for 30 s) using (a) -314 AGT for (CTCAGGCTGTCACACACCTA) as a forward and -6AGT rev (TCTTCCCCTGGCCGGGTCACGAT) as a reverse primer, when GR and CEBPβ antibodies were used to examine their binding at the -217 position of the hAGT gene, or (b) -1757 AGT for (CAGGCACAGTGGAAACTCTCC) as a forward primer and -1554 AGT rev (AGTAACAAGTCCACCTGGAC) as a reverse primer, when GR and CEBPβ antibody were used to examine the binding of GR across the -1561 and -1562 polymorphic sites of the hAGT gene. The PCR-amplified products were analyzed on 2% agarose gel. The relative band intensities by densitometry were measured using Quantity One quantitation software from Bio-Rad, and the results were normalized to the band intensities from the respective input DNA. The fraction enriched by rabbit IgG was used as a negative control for nonspecific binding. A primer set that amplified the DNA fragment of the hAGT gene with no known glucocorticoid response element was used as a nonspecific control for GR-enriched DNA fragments. In another set of experiments, 900ul of chromatin solution was immunoprecipitated with pSTAT3 antibody. Immunoprecipitated DNA was then amplified using CTCAGGCTGTCACACACCTA as a forward primer and (CTTTATGTTTTTGGCGTCTTCCA) (Promega Biotech) a reverse primer to amplify APRE-1(Acute phase response elements; STAT binding site) regions. Oligonucleotide TATGCTAGGGCTCACTCTGTTCAGCAGTGAAACTC was used as a forward primer and the reverse primer for amplification of APRE-3 (STAT binding site) region in the hAGT proximal promoter region. Oligonucleotide GAGGTATTTGTGTGTTTGTTGATTGT and ACAGGGCATGACAGAGACCTTGG were used as forward and reverse primers to amplify 320 bp (located between -770 and -1,093) of the upstream region of the hAGT promoter.

### Transfac analysis

In silico analysis of the hAGT promoter and transcription factor binding sites is performed using TRANSFAC (TRANScription FACtor database) analysis. The Transfac web site was used in our analysis mentioned in this paper but this site has been dis-continued. We are now using www.gene-regulation.com/pub/programs.html for analysis of the transcription factor binding sites.

### Statistical analyses

All experiments were conducted with four animals in each group. Data are expressed as the means S.E. Statistical significance was assessed using two-way analysis of variance with a Tukey-Kramer post hoc analysis. Two factors for the two-way ANOVA are, haplotype and diet. The significance level was set at (p≤ 0.05). For Figs 1A and 1B and 4A and 4B, unpaired T-test is used to assess the null-hypothesis.

## Supporting information

S1 Fig Quantitative RT-PCR analysis of hAGT and mAGT mRNA level in adipose and liver tissues. Human AGT expression was significantly elevated after high fat diet (HFD) in TG mice with Hap I compared to Hap II in adipose and liver tissue (1A, 1B). Figure shows the quantitative RT-PCR analysis of hAGT and mAGT mRNA level in adipose (A, C) and in liver (B, D) in TG mice after 12 weeks of control diet or HFD. n = 4 per group in both CD and HFD groups.
(TIF)

S2 Fig Differential expression of transcription factors GR, CEBPβ and STAT3 mRNA in adipose and liver of TG mice after CD or HFD. Relative mRNA expression is calculated for each group compared with its respective control diet group (n = 4).
(TIF)

S3 Fig Relative binding of transcription factors GR, CEBP and STAT3 to the promoter of hAGT gene. Q-PCR was performed to quantify the relative binding at -217 region. ChIP assay was performed from the chromatin obtained from the adipose tissue of HFD treated TG animals (n = 4).
(TIF)

S4 Fig Relative binding of transcription factors GR and CEBP to the promoter of hAGT gene. Q-PCR was performed to quantify the relative binding at -1329 region. ChIP assay was performed from the chromatin obtained from the adipose tissue of HFD treated TG animals (n = 4).
(TIF)

S5 Fig Plasma levels of hAGT in TG mice containing either Hap I or Hap II of the hAGT gene in CD or HFD fed TG mice. Plasma AGT levels were determined by an ELISA (n = 4).
(TIF)

S1 File File containing individual data points for Figs 1–5. (XLSX)
