# Supplementary figures and images for: The transcriptional regulation of the human angiotensinogen gene after high-fat diet is haplotype-dependent: Novel insights into the gene-regulatory networks and implications for human hypertension
Source: PLoS One. 2017 May 3;12(5):e0176373. doi: 10.1371/journal.pone.0176373 (PMC5415177; doi:10.1371/journal.pone.0176373)

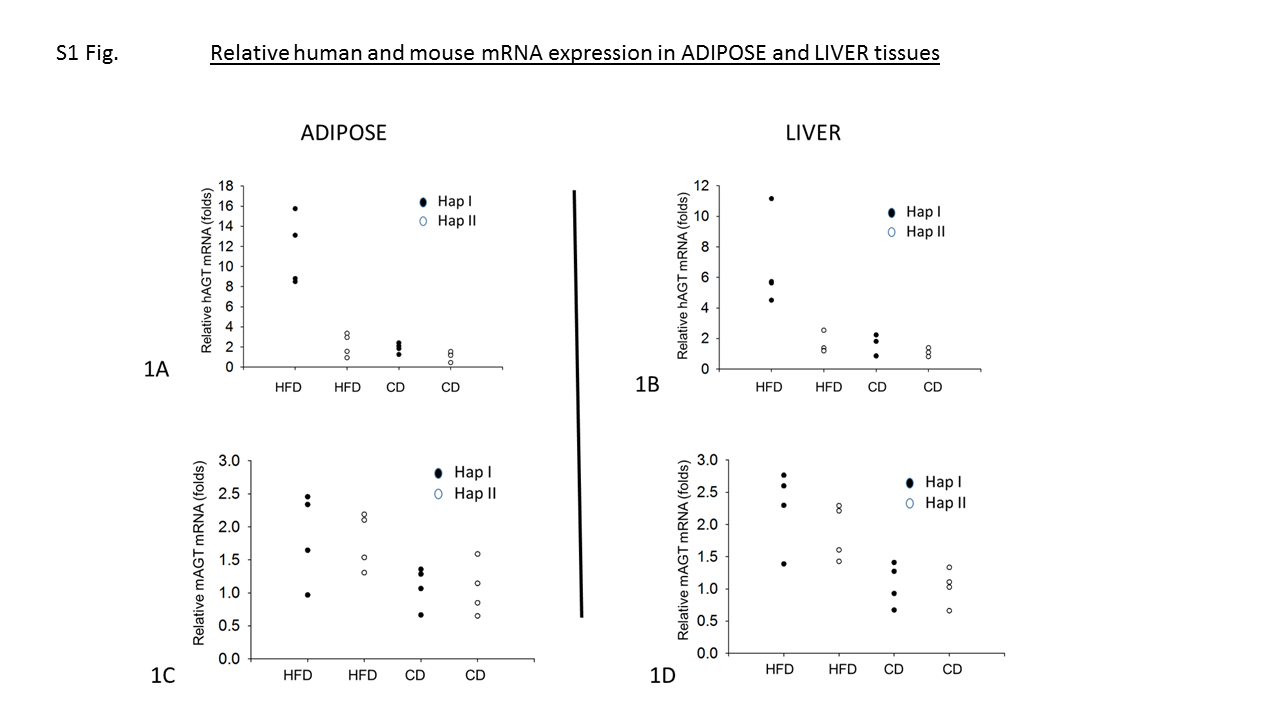

Supplement: S1 Fig — Human AGT expression was significantly elevated after high fat diet (HFD) in TG mice with Hap I compared to Hap II in adipose and liver tissue (1A, 1B). Figure shows the quantitative RT-PCR analysis of hAGT and mAGT mRNA level in adipose (A, C) and in liver (B, D) in TG mice after 12 weeks of control diet or HFD. n = 4 per group in both CD and HFD groups. (TIF) [file pone.0176373.s001.TIF]

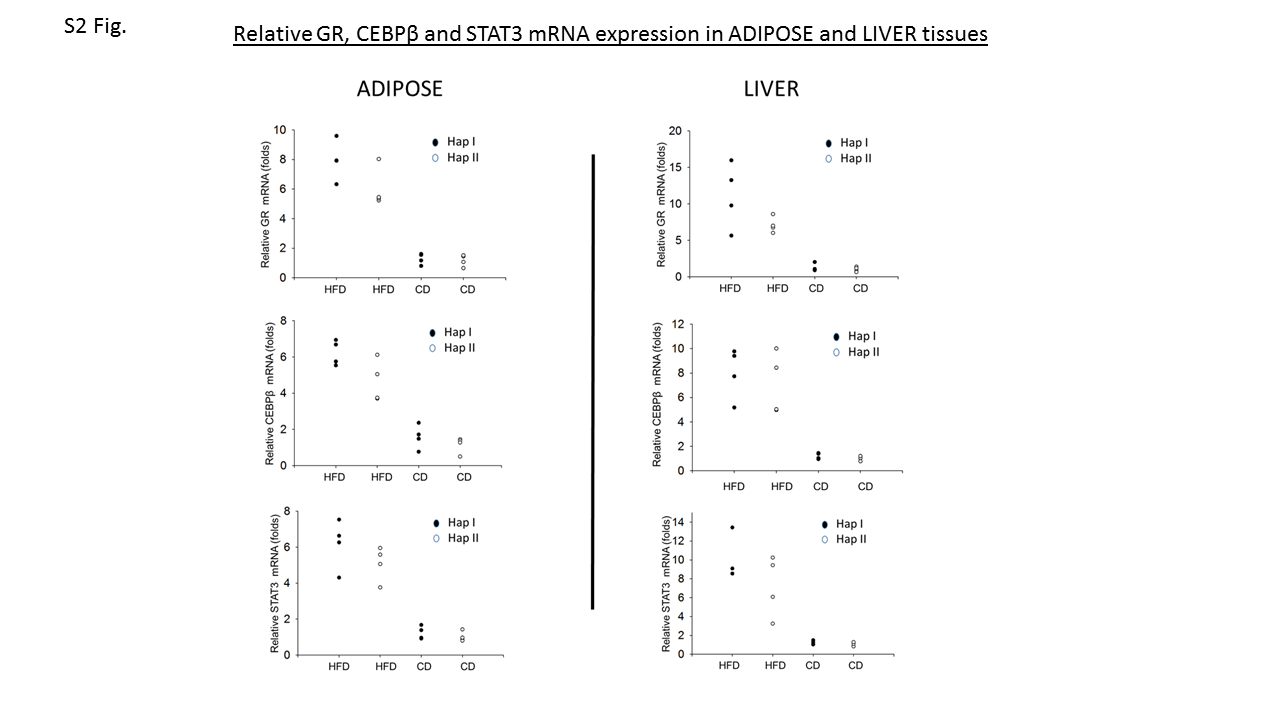

Supplement: S2 Fig — Relative mRNA expression is calculated for each group compared with its respective control diet group (n = 4). (TIF) [file pone.0176373.s002.TIF]

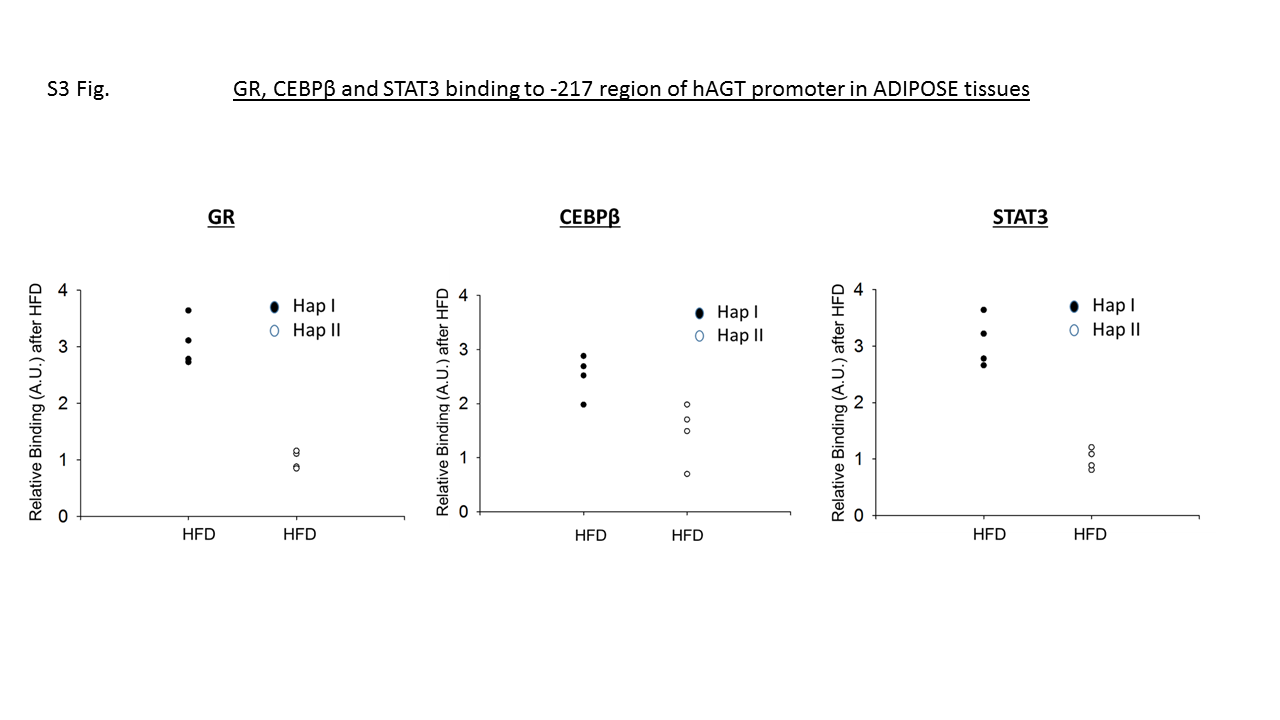

Supplement: S3 Fig — Q-PCR was performed to quantify the relative binding at -217 region. ChIP assay was performed from the chromatin obtained from the adipose tissue of HFD treated TG animals (n = 4). (TIF) [file pone.0176373.s003.TIF]

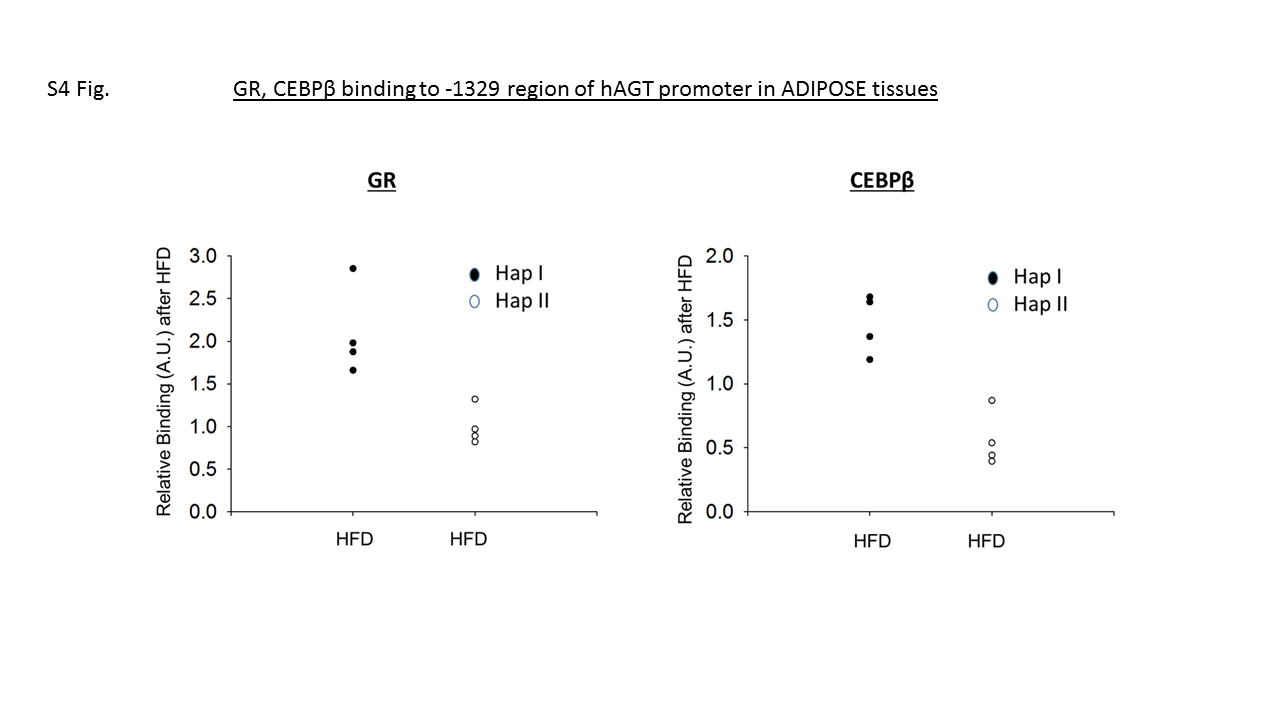

Supplement: S4 Fig — Q-PCR was performed to quantify the relative binding at -1329 region. ChIP assay was performed from the chromatin obtained from the adipose tissue of HFD treated TG animals (n = 4). (TIF) [file pone.0176373.s004.TIF]

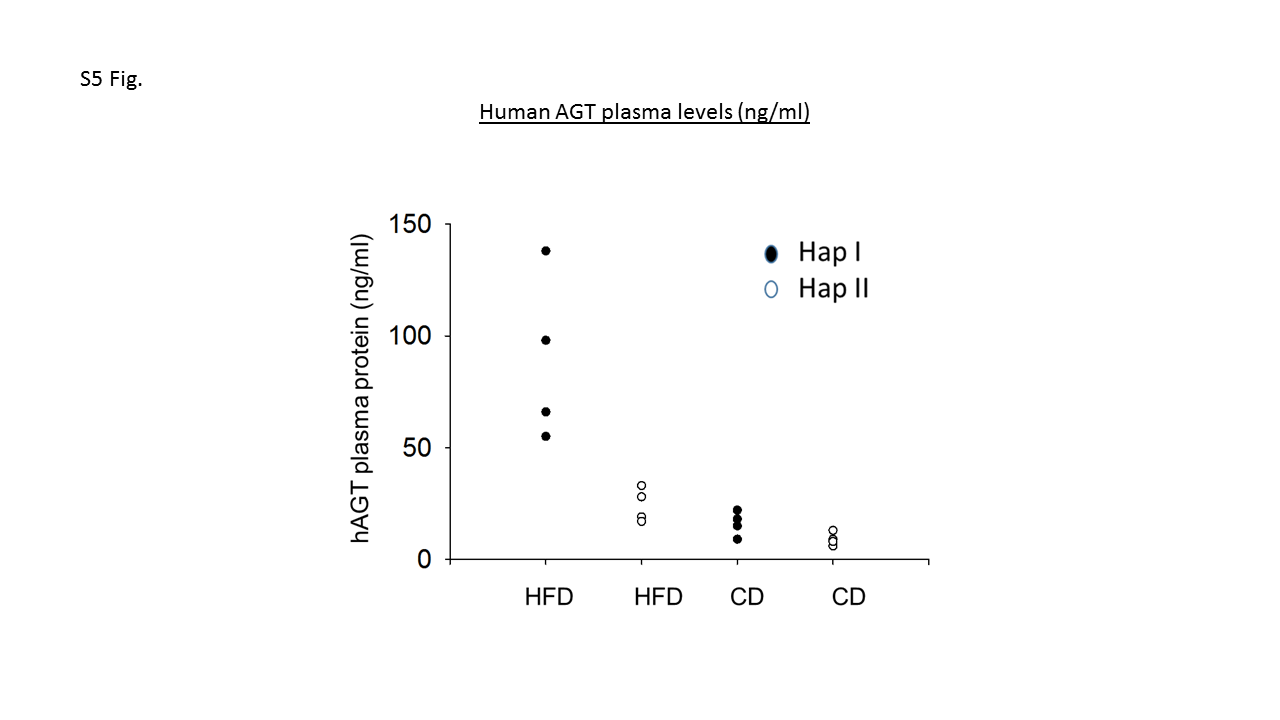

Supplement: S5 Fig — Plasma AGT levels were determined by an ELISA (n = 4). (TIF) [file pone.0176373.s005.TIF]
